# Body Mass Index and Dental Caries: A Systematic Review

**DOI:** 10.5005/jp-journals-10005-1516

**Published:** 2018-06-01

**Authors:** Sahana Shivakumar, Adit Srivastava, Ganiga C Shivakumar

**Affiliations:** 1 Reader, Department of Public Health Dentistry, Babu Banarasi Das College of Dental Sciences, Lucknow, Uttar Pradesh, India; 2 Associate Professor, Unit of Oral Medicine and Radiology, Department of Oral Medicine & Radiology, Faculty of Dental Sciences, Institute of Medical Sciences, Banaras Hindu University, Varanasi, Uttar Pradesh, India; 3 Professor, Department of Oral Medicine and Radiology, Babu Banarasi Das College of Dental Sciences, Lucknow, Uttar Pradesh, India

**Keywords:** Association, Body mass index, Cross-sectional study, Dental caries, Review.

## Abstract

**Introduction:**

This review was undertaken to analyze the relationship between body mass index (BMI) and dental caries with the available literature evidence.

**Materials and methods:**

The articles were searched from Medline/PubMed and Journal of Web published between 2005 and 2016.

**Results:**

Out of the 146 references obtained, 16 articles in English language were read in full, which fulfilled the inclusion criteria after assessing by Down and Black criteria.

**Conclusion:**

No consensus was reached in the relationship between BMI and dental caries in the present review due to varied associations

**How to cite this article:** Shivakumar S, Srivastava A, Shivakumar GC. Body Mass Index and Dental Caries: A Systematic Review. Int J Clin Pediatr Dent 2018;11(3):228-232.

## INTRODUCTION

Oral diseases, especially dental caries, are still mainly prevalent in most developing countries, affecting people from all races, socioeconomic status, and ages. This disease, dental caries, still continues to be a public health problem in spite of technological advancements and a better understanding of the carious process.^1^ Dental caries is a multifactorial disease attributed to both modifiable risk factors like dietary factors, water fluoride levels, tooth brushing frequency, and nonmodifiable risk factors like socioeconomic status and previous caries experience. The focus now is shifted to modifiable factors, specifically diet, in the prevention of dental caries.

Body mass index is an anthropometric measurement which measures weight relative to the height. Though it is often used to estimate the level of body fat in individuals, it provides an excellent indicator of obesity-related health risks.

The world is witnessing an increasing number of overweight individuals owing to the consumption of fast food and soft drinks coupled with lack of activity and exercise. Overweight individuals are associated with prolonged exposure to carbohydrates.^2^ Excessive consumption of refined carbohydrates, especially sugar in its refined form, is associated both with dental caries and being overweight and obese.^3^ An association between BMI and dental caries works probably on this possibility. The Scientific Advisory Committee on Nutrition^4^ (London) reported an association of higher consumption of free sugars with dental caries. The consumption of sugar-sweetened beverages further leads to greater weight gain and increase in BMI.

Literature provides evidence for the coexistence of obesity and dental caries, as they have common risk factors like consumption of free sugars and socioeconomic deprivation. Overweight and dental caries are attributed to complex behavioral and societal factors which include genetic component, increased media exposure through television and computer games, overall calorie intake along with increased intake of sugary foods and beverages, physical activity, habits of both oral hygiene and personal. Various literature stands evidence to the coexistence of the two conditions in the same individuals and populations, but with variations.^5-7^ The review hence, was undertaken with the objective of establishing a scientific relationship between BMI and dental caries.

## MATERIALS AND METHODS

The literature search of published articles was performed in the electronic databases of the Medline/PubMed, and Journal of Web, between 2005 and 2016. Only study articles which investigated the relationship between dental caries and BMI on all ages were looked for, excluding systematic reviews and meta-analysis published. The terms used for literature search were dental caries and BMI. Two independent researchers searched the databases and identified 68 relevant studies (Flow Chart 1).

**Flow Chart 1:**
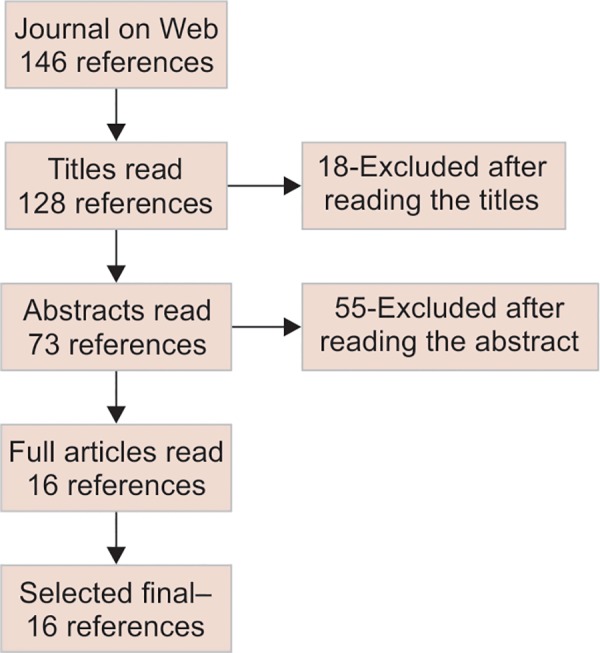
Literature search flow chart for systematic review on BMI and dental caries

Articles published before 2004, studies done on specific groups, and dissertations were excluded. Downs and Black^8^ criteria were used to assess the quality of scientific evidence in chosen articles. Out of the 27 items criteria, 10 had to be excluded, as it could be applied only for intervention studies and none of the study chosen had an experimental study design. Studies which scored more than 10 items criteria were considered of good scientific evidence. This criterion of fulfilling 50% or greater items of the Downs and Black^8^ criteria was set by the study authors. No cases of disagreement were reported between the authors for any of the studies researched.

## RESULTS

A total number of 146 reference articles were obtained in Journal of Web, out of which 18 articles were excluded after reading the titles. Out of this, 55 articles were excluded after reading the abstracts, making the count to 73. Finally, 16 full articles in English were included in the review, which met the inclusion criteria of the reviewers.

Table 1 shows the list of studies reviewed on BMI and dental caries. The review presented contradictory reports. While studies conducted by de Jong-Lenters et al,^9^ Lempert et al,^10^ Sharma et al,^11^ Martins et al,^12^ Chukwumah et al,^13^ Elangovan et al,^14^ Cinar and Mur-tomaa,^15^ and Pinto et al^16^ did not report any association between BMI and dental caries, the studies of Creske et al,^17^ Bagherian and Sadeghi,^18^ Shahraki et al,^19^ Can-tekin et al,^20^ Thippeswamy et al,^21^ and Willerhausen et al^22^ reported a significant association between BMI and dental caries. Studies of Shailee et al^23^ and Parkar and Chokshi^24^ revealed a negative correlation between Decayed, extracted, filled teeth (deft) and BMI. Among the 16 studies reviewed, 14 studies employed a cross-sectional design, one prospective cohort structure, and one study a case-control design as mentioned in Table 1.

## DISCUSSION

The present review tried to analyze a relationship between BMI and dental caries based on articles published from the period between 2005 and 2016. The results of this systematic review suggest that no consensus has been reached in the various studies included because of varied associations. Out of the 73 abstracts read, 55 were excluded, making the final selected articles to a total of 16. Of the 16 articles reviewed, 14 were cross-sectional studies, 1 case-control study, and 1 prospective cohort study.

The standard way of recording the anthropometric assessment of BMI is done by using a 150 kg digital scale and 200 cm tape to measure height according to the World Health Organization (WHO) guidelines. The body weight was recorded by using a standard beam balance scale with participants wearing light dresses and barefoot. Body height is recorded with subjects not wearing any shoes and head touching the ruler with line of sight aligned horizontally. The BMI is calculated by the formula: Weight (kg)/height (m^2^). The interpretation of the scores are underweight (<18.5), normal weight (18.5-24.99) and overweight (>25) as per WHO.^25^ The ease of implementation and objectivity makes BMI a popular tool to measure obesity. But, this index is to be considered cautiously, as it can produce false-positive results for the fact that it cannot differentiate between lean body mass and fat mass.

Dental caries diagnosis in most of the studies employed visual examination of the exposed teeth or surfaces. The DEFT index was recorded to measure dental caries in primary dentition and decayed, missing, filled teeth (DMFT) index was used to record dental caries in permanent dentition. But, this method of examination leads itself to underreporting of the disease. Use of interproximal radiographs is more sensitive to caries diagnosis, but not very suitable for epidemiological surveys, as it is both expensive and increases the risk of radiation exposure.

Systematic reviews conducted by several authors^26^ excluded children under 6 years of age, considering the observation of increased intake of cariogenic food in this age group, which poses a significant risk to both dental caries and obesity. Also, parental influence and super-vision on this age group decide the diet pattern, and oral hygiene practices dictate caries prevalence. While parental control on sugar consumption can lower caries experience, lack of control can even increase the chance of caries and, subsequently, obesity. Not excluding any population for the review was in accordance with the study of Kantovitz et al.^27^

**Table 1:** **Table 1**: Review of studies on BMI and dental caries

*Authors*		*Year*		*Study site*		*Age group*		*size*		*Study design*		*Results*	
de Jong-Lenters et al^9^		2015		Pediatric dental care in Noordojk, the Netherlands		5-8 years		230		Cross-sectional		Results showed no statistically significant differences between the mean DMFT or decayed missing filled surface (DMFS) scores of overweight and nonoverweight children, even after adjusting for potential confounders like gender, socioeconomic status and ethnicity	
Lempert et al^10^		2014		Data from European Youth Heart Study and Danish National Board of Health		9.6 years		385		Case-control study		No significant association was found between caries experience and BMI	
Sharma et al^11^		2014		Meerut district, India		13-17 years		504		Cross-sectional		The association between BMI and caries was statistically nonsignificant with p-value 0.661 even when both genders were analyzed separately	
Creske etal^17^		2013		Riverside County’s Coachelle valley		6-11 years		177		Cross-sectional		Results showed that children in the obese category had a statistically significant lower rate of DMFT than the children of healthy weight category	
Martins etal^12^		2013		Charity institution inAracatuba, Brazil		3.9 ±1.0		91		Cross-sectional study		Contingency C coefficient test found no association between BMI and caries	
Bagherian and Sadeghi^18^		2013		Rafsanjan, Iran		30-70 months		400		Cross-sectional		The results revealed a statistically significant direct association between BMI for age and dental caries (p = 0.001), after adjusting for gender and	
Shahraki etal^19^		2013		Zahedan, Iran		6-11 years		1213		Cross-sectional		Results revealed a significant association between BMI and DFT (p = 0.005). BMI for age values revealed that 34 children were caries-free in the normal weight and underweight cases, while 28 children in the overweight and obese groups were caries-free	
Shailee etal^23^		2013		Shimla city, India		12 and 15 years		1011		Cross-sectional study		Results showed a negative correlation of BMI with DMFT (r = 0.312, p < 0.011)	
Parkarand Chokshi^24^		2013		Ahmedabad city, India		10.96 + 3.14 years		750		Cross-sectional study		A negative correlation was observed between deft and BMI, which was significant	
Chukwumah et al^13^		2012		Ugbowo, Benim city, Nigeria		7-15 years		210		Cross-sectional		There was no significant association between BMI and caries experience	
Elangovan et al^14^		2012		Private dental college, Tamil Nadu, India		6-12 years		510		Cross-sectional study		There was no statistically significant difference in the mean caries score between children belonging to various BMI for age categories (p > 0.05)	
Cantekin et al^20^		2012		Erzurum, Turkey		12 years		224		Cross-sectional study		A possible correlation between obesity and caries was seen, but not between overweight and caries	
Thippeswamy et al^21^		2011		Udupi, South India		13-15 years		463		Cross-sectional		Analysis revealed that obese group of children had more caries than the overweight and normal weight children. Correlation analysis showed significant positive relation with BMI	
Cinarand Murtomaa^15^		2008		Finland and Turkey		10-12 years		338 + 611		Cross-sectional		No association was seen between BMI and dental caries in both crude and adjusted analysis	
Pinto etal^16^		2007		Pediatric dental clinic of Pennsylvania School of Dental Medicine		8.7 ±2.37		135		Prospective cohort study		No correlation was found between dental decay in obese and nonobese children (p = 0.99)	
Willerhausen et al^22^		2007		Germany		6-11 years		1298		Cross-sectional study		A positive statistical association was observed between BMI and caries in both deciduous and permanent dentition	

The review included studies published in English language only, incorporating an element of selection bias. The variation in the study designs and reporting reflected on the quality of the included articles. Most of the studies reviewed did not consider the various confounding variables like socioeconomic factors, dietary pattern, and oral hygiene practice which could have a played a major role in the establishment of a relationship. Factors to be considered in addition to the above confounders are utilization of oral health services and use of fluoridated substances. These are potential effect modifiers which may result in a weak or negative association between BMI and dental caries. The Downs and Black^8^ instrument used to assess the quality of studies was also employed by other authors^26^ because of its clarity. Though it was originally designed to evaluate intervention studies, it can be employed in observational studies also after excluding certain items.

## CONCLUSION

No agreement was reached on the relationship between BMI and dental caries because of varied associations of the studies reviewed and for not including the effect of confounders and effect modifiers. It is recommended to conduct newer and clearly delineated studies in future to provide valuable clues regarding this relationship.
